# HIV experts on the decision to use early ART for prevention in France: are we there yet?

**DOI:** 10.1186/1742-4690-9-S1-P95

**Published:** 2012-05-25

**Authors:** Bertrand Lebouché, Kim Engler, Joseph Josy Lévy, Gimore, Bruno Spire, Willy Rozenbaum, Jean-Pierre Routy

**Affiliations:** 1 Mcgill University Health Centre, Montreal, Canada; 2 Université du Québec à Montréal, Canada; 3 INSERM-SESSTIM UMR912, Marseille, France; 4 Hôpital Saint-Louis, Paris, France

## Introduction

The finding that successful antiretroviral therapy (ART) can almost eliminate the risk of heterosexual HIV transmission is the scientific breakthrough of 2011 according to Science Magazine. This potential of ART has generated novel approaches to prevention including “Test and Treat” (T&T). Our qualitative study, drawing on the perspectives of French HIV experts, aims to better understand if, and how, a T&T approach might be applied in France and to generally learn more about concerns raised by prevention with early ART.

## Materials and methods

In 2011, 19 French HIV experts participated in a semi-structured interview on implementing T&T in France. Expertise was typically defined on the basis of contribution to the 2010 French ART guidelines (Rapport Yeni 2010). Participants’ HIV expertise included clinical care, epidemiology, virology, and community activism. Interviews lasted an hour, on average, and explored opinions on T&T, who, how and whether to test and treat, and what public health discourses and evaluations should accompany it. Analyses of interview content pertaining to who or whether to treat early for prevention are presented here. A content analysis of the transcribed interviews was supported by the software Atlas.ti version 5.2.

## Results

The decision to treat earlier than current guidelines recommend (CD4 >500) was generally associated with uncertainties and involved weighing the risks and benefits, whether potential or known, primarily in terms of patient health, the risk of transmission, patient choice, population health and/or cost. Perspectives on each of these aspects varied as did experts' position on treating early for prevention.

## Conclusions

Despite ART-based HIV prevention’s status as evidence based medicine (EBM), French experts had uncertainties about early preventive treatment of HIV-infection which often translated into a weighing of benefits and risks to varying effect. Our findings suggest fostering a culture of dialogue and debate between care providers, community organizations and recipients of care on the complex considerations of implementing strategies of ART as prevention in France can help translate EBM into improvements in health and new HIV guidelines towards the eradication of HIV.

